# Gut health of horses: effects of high fibre vs high starch diet on histological and morphometrical parameters

**DOI:** 10.1186/s12917-022-03433-y

**Published:** 2022-09-08

**Authors:** Elena Colombino, Federica Raspa, Maria Perotti, Domenico Bergero, Ingrid Vervuert, Emanuela Valle, Maria Teresa Capucchio

**Affiliations:** 1 Department of Veterinary Sciences, University of Turin, Largo Paolo Braccini 2, Grugliasco, 10095 Turin, Italy; 2 Institute of Animal Nutrition, Nutrition Diseases and Dietetics, Faculty of Leipzig, University of Leipzig, Leipzig, 04103 Germany

**Keywords:** Gut health, Welfare, Nutrition, Morphometry, Starch, Fibre,

## Abstract

**Background:**

The conventional feeding management of horses is still characterized by high starch and low fibre diets, which can negatively affect horse’s gastrointestinal health. Thus, the aim of this study was to compare the effects of a high-starch (HS) vs. a high-fibre (HF) diet on gut health in horses. A total of 19 Bardigiano horses destined for slaughter and aged 14.3 ± 0.7 months were randomly allotted to two dietary groups: HS (5 fillies and 4 colts,) and HF group (7 fillies and 3 colts). They received the same first-cut meadow hay but different complementary feeds for 72 days: HS group was fed 8 kg/animal/day of a starch-rich complementary feed while HF group was fed 3.5 kg/animal/day of a fibre‐rich complementary feed. At slaughter, stomachs were separated and washed for the evaluation of the glandular and squamous regions. Also, duodenum, jejunum, ileum, apex of the caecum, sternal flexure, pelvic flexure, right dorsal colon, rectum and liver were excised and submitted to histomorphometrical evaluation.

**Results:**

The glandular region of HS group presented more severe gastric mucosa lesions compared to the HF group (*P* = 0.006). Moreover, a statistical tendency (*P* = 0.060) was found for the squamous region, presenting a higher score in HS than HF diet. Regarding morphometry, in jejunum villus height to crypt depth (Cd) ratio was influenced by sex, being greater in males than in females (*P* = 0.037) while in ileum Cd depended on interaction between sex and diet, being greater in males of HS group (*P* = 0.029). Moreover, in the duodenum and right dorsal colon the severity of the inflammation depended on sex (*P* = 0.024 and 0.050), being greater in females than in males. On the contrary, in the jejunum and in the pelvic flexure, inflammation was influenced by diet, being more severe in HS than in HF group (*P* = 0.024 and 0.052).

**Conclusions:**

These results suggested that HS diet provoked more severe mucosa lesions in the glandular region of the stomach and a higher inflammation both in the jejunum and pelvic flexure. The present study can represent a starting point for further investigations on gut health in horses.

## Background

Feeding horses energy-dense feedstuffs which contain significant amounts of starch is still a common mistake in feeding practice for athletic or leisure horses [1]. Moreover, this feeding management is conventional in intensive breeding farms that rear horses for meat production [2]. In particular, it is reported that horses reared for meat production are primarily young horses of several breeds (e.g. Heavy French breeds, Italian Heavy Draft horses, Bardigiano) slaughtered between 11 and 18 months of age [3–5]. Due to the need of obtaining a faster increase in body weight in a shorter fattening period, those animals are mainly reared in intensive farming system with a high stock density and with a high-starch and low-fibre diet [2]. However, feeding high starch diets to horses is in conflict with their natural feeding behaviour and it can negatively affect their health, welfare and performances [6].

Horses are non-ruminant herbivores well suited to a high fibre, low starch diet that would naturally spend up to 18 h/day foraging and rarely fast for more than 2–4 h at a time [7]. They are hindgut fermenters, with an extensive and complex caecum and large colon suitable for fermentation of fibre structural carbohydrates. On the contrary, the upper part of the gastrointestinal tract has a limited ability to digest high amounts of starch as a consequence of the limited production of the pancreatic amylase. For these reasons, large cereal grain meals (> 2 gr of starch/kg bodyweight/meal) may overwhelm the digestive capacity of the small intestine, leading to the rapid fermentation of the indigested starch in the hindgut [6]. This can potentially result in an impairment of gastrointestinal health, causing dysbiosis, colic and gastric ulcers [8].

Gastrointestinal health is crucial in veterinary practice and it can be considered synonymous with animal’s health [9]. In fact, gut health depends on effective digestion and absorption of feed, proper structure of the gastrointestinal barrier, absence of gastrointestinal illness, normal and stable intestinal microbiota, and effective immune status [10]. If gut health is compromised, digestion and nutrient absorption are affected with a detrimental effect on animal growth performances, leading to economic loss and greater susceptibility to disease [11]. In particular, the diet has been proven to be one of the most important factors in influencing gut health [9, 12]. Accordingly, fast dietary changes and excessive amounts of cereals grains in the diet have been well-recognized as important stressors for the gastrointestinal system of the horse. Feeding horses a high concentrate diet causes a poorly buffered and acid environment in the stomach, increasing the incidence and severity of gastric ulceration [13, 14]. Moreover, when the indigested starch reaches the hindgut, it can causes an increase in lactic acid–producing bacteria which are associated with a higher risk of colic [15, 16]. It is also reported that this increase in lactic acid production and the subsequent acidosis cause important damage to the intestinal epithelium. In fact, Stewart et al., [12] stated that acidosis can lead to a condition of intestinal hyperpermeability, also known as “leaky gut”. However, to the authors’ knowledge, no studies have investigated if a starch-rich diet can affect intestinal histomorphology through the modifications of villus height and crypt depth in horses. Moreover, it has been shown that hyperpermeability could be associated with increased bacterial contamination of mesenteric lymph nodes and liver [4].

On this basis, the aim of the present study was to compare the effects of a high-starch vs. a high-fibre diet on several aspects of gut health in horses: severity of gastric mucosa lesions, gut histomorphometry and liver histology.

## Results

### Growth performances

At the end of the study, the mean ± SD slaughter Body Weight (sBW) of horses in HS group was 347.67 ± 6.71 kg; whereas the sBW of horses in HF group was 344.40 ± 2.91 kg. Moreover, the mean ± SD daily bodyweight gain was 1.01 ± 0.06 kg in HS group and 0.95 ± 0.05 kg in HF group.

### Stomach macroscopical evaluation

**Fig. 1:**
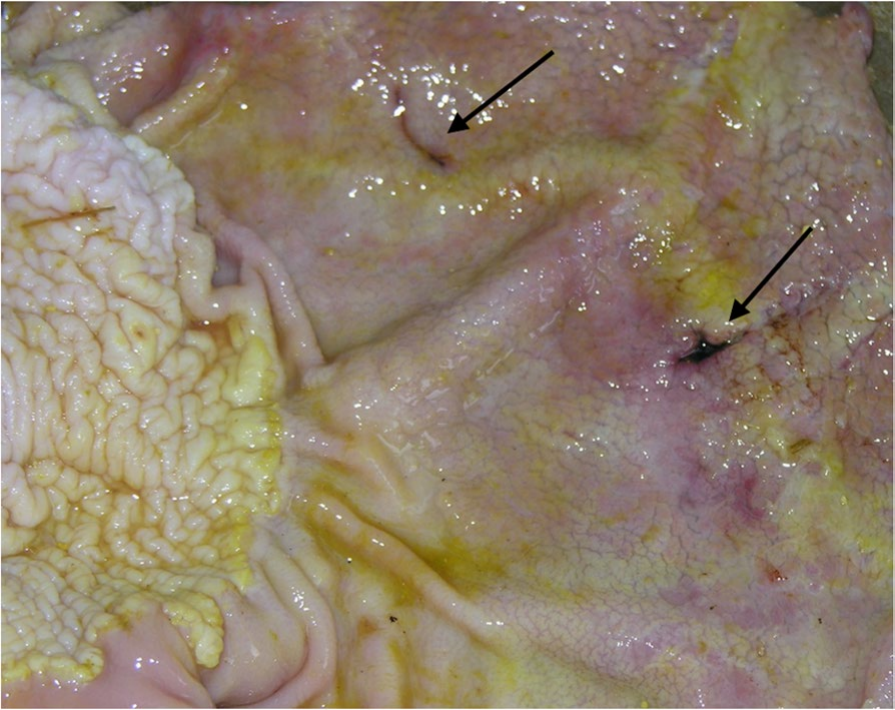
Glandular region of the stomach from a horse fed the high starch diet (HS) showing gastritis with multiple ulcerations

Data regarding stomach macroscopical evaluation were summarized in Table 1. The glandular region of horses in HS group presented more severe gastric mucosa lesions compared to those seen in horses belonging to the HF group (*P* = 0.006) (Fig. 1). Moreover, the squamous region showed a statistical tendency (*P* = 0.060), presenting a higher score in HS than in HF diet.

**Table 1:** Stomach lesion grades of the horses analysed in the present study (*n* = 19)

Variable	Diet (D)		Sex (S)		*P*-value		
	HS	HF	Female	Male	D	S	DxS
Glandular region, *mean (SD)*	2.167^a^ (1.17)	0.400^b^ (0.69)	1.227 (1.13)	1.250 (1.58)	0.006	0.446	0.980
Squamous region, *mean (SD)*	1.556 (0.73)	0.800 (1.13)	1.091 (1.04)	1.250 (1.03)	0.060	0.955	0.322

*HS* High-starch diet, *HF* High-fibre diet

Regarding sex or interaction between diet and sex, it did not influence the severity of gastric lesions both in the glandular and squamous regions (*P* > 0.05).

### Morphometrical and histopathological investigations

As reported in Table 2, non-significant differences were recorded for all the evaluated morphometrical indices in duodenum (*P* > 0.05). On the contrary, in jejunum villus height (Vh) to crypt depth (Cd) ratio was influenced by sex, being greater in males than in females (*P* = 0.037) while in ileum Cd depended on the interaction between sex and diet, being greater in males of HS group (*P* = 0.029, Table 2).

**Table 2:** Morphometrical indices of the horses analysed in the present study (*n* = 19)

Variables (mm)	Diet (D)		Sex (S)		*P*-value		
	HS	HF	Female	Male	D	S	Dxs
**Duodenum**							
Villus height (Vh), *mean (SD)*	0.393 (0.07)	0.364 (0.07)	0.383 (0.09)	0.370 (0.03)	0.324	0.702	0.711
Crypt depth (Cd), *mean (SD)*	0.127 (0.02)	0.051 (0.01)	0.113 (0.19)	0.051(0.006)	0.352	0.351	0.340
Vh/Cd ratio, *mean (SD)*	6.919 (1.30)	6.796 (1.43)	6.616 (1.39)	7.181 (1.26)	0.965	0.442	0.689
Villus width, *mean (SD)*	0.184 (0.53)	0.173 (0.02)	0.187 (0.05)	0.166 (0.02)	0.420	0.323	0.984
Mucosa thickness, *mean (SD)*	0.664 (0.11)	0.645 (0.08)	0.656 (0.11)	0.650 (0.06)	0.696	0.864	0.497
Villus absorptive area, *mean (SD)*	0.224 (0.06)	0.200 (0.06)	0.224 (0.07)	0.194 (0.03)	0.202	0.255	0.732
**Jejunum**							
Vh, *mean (SD)*	0.372 (0.03)	0.417 (0.09)	0.386 (0.09)	0.410 (0.05)	0.118	0.159	0.465
Cd, *mean (SD)*	0.049 (0.10)	0.050 (0.007)	0.058 (0.01)	0.088 (0.11)	0.099	0.074	0.347
Vh/Cd ratio, *mean (SD)*	6.669 (2.95)	8.453 (2.10)	7.350^a^ (2.10)	7.962^b^ (3.35)	0.079	0.037	0.986
Villus width, *mean (SD)*	0.187 (0.02)	0.195 (0.03)	0.194 (0.03)	0.188 (0.01)	0.989	0.926	0.349
Mucosa thickness, *mean (SD)*	0.689 (0.07)	0.685 (0.11)	0.677 (0.11)	0.699 (0.07)	0.759	0.275	0.279
Villus absorptive area, *mean (SD*)	0.219 (0.03)	0.261 (0.10)	0.241 (0.10)	0.241 (0.03)	0.184	0.189	0.846
**Ileum**							
Vh, *mean (SD)*	0.438 (0.04)	0.430 (0.07)	0.435 (0.06)	0.432 (0.05)	0.696	0.995	0.821
Cd, *mean (SD)*	0.054 (0.01)	0.050 (0.01)	0.053 (0.01)	0.051 (0.01)	0.222	0.462	0.029
Vh/Cd ratio, *mean (SD)*	8.100 (1.11)	8.858 (2.27)	8.437 (2.12)	8.583 (1.41)	0.560	0.627	0.254
Villus width, *mean (SD)*	0.186 (0.03)	0.202 (0.02)	0.195 (0.02)	0.194 (0.03)	0.321	0.453	0.196
Mucosa thickness, *mean (SD)*	0.751 (0.11)	0.769 (0.16)	0.773 (0.16)	0.743 (0.10)	0.814	0.834	0.175
Villus absorptive area, *mean (SD)*	0.259 (0.06)	0.275 (0.06)	0.267 (0.05)	0.268 (0.07)	0.773	0.700	0.629

*HS* High-starch diet, *HF* High-fibre diet

 Regardless of dietary treatment, multifocal to diffuse lymphoplasmacytic enteritis of variable severity with or without lymphoid tissue activation was recorded in all the examined gut segments with a proximo-distal increasing gradient from duodenum to cecum (Table 3). Particularly, in the duodenum and right dorsal colon the severity of the lesions depended on sex (*P* = 0.024 and 0.050, respectively), being greater in females than in males (Table 3).

**Table 3:** Histopathological evaluation of gut segments and liver of the horses of the present study

Variables	Diet (D)		Sex (S)		*P*-value		
	HS	HF	Female	Male	D	S	DxS
Inflammation duodenum, *mean (SD)*	1.722 (0.79)	1.400 (0.57)	1.727^a^ (0.65)	1.312^b^ (0.70)	0.216	0.024	0.523
Inflammation jejunum, *mean (SD)*	1.889^a^ (0.55)	1.400^b^ (0.66)	1.773 (0.75)	1.438 (0.42)	0.008	0.143	0.345
Inflammation ileum, *mean (SD)*	3.222 (1.28)	2.500 (1.35)	3.182 (1.63)	2.375 (0.58)	0.091	0.147	0.348
Inflammation cecum, *mean (SD)*	4.889 (0.48)	4.600 (0.97)	4.909 (0.77)	4.500 (0.75)	0.399	0.276	0.854
Inflammation sternal flexure, *mean (SD)*	2.889 (0.99)	2.550 (0.50)	2.773 (0.93)	2.625 (0.52)	0.425	0.468	0.726
Inflammation pelvic flexure, *mean (SD)*	3.222 (0.67)	2.800 (0.75)	3.182 (0.72)	2.750 (0.71)	0.052	0.092	0.907
Inflammation right dorsal colon, *mean (SD)*	3.167 (1.06)	3.000 (1.07)	3.138^a^ (0.95)	2.250^b^ (0.96)	0.126	0.050	0.718
Inflammation rectum, *mean (SD)*	3.000 (1.03)	2.400 (0.77)	2.773 (1.15)	2.562 (0.56)	0.111	0.617	0.987
Inflammation liver, *mean (SD)*	1.056 (0.30)	1.000 (0.23)	0.954 (0.27)	1.125 (0.23)	0.880	0.320	0.320
Degeneration liver, *mean (SD)*	1.778 (0.67)	2.150 (0.78)	1.818 (0.68)	2.188 (0.80)	0.155	0.206	0.456

*HS* High-starch diet, *HF* High-fibre diet

**Fig. 2:**
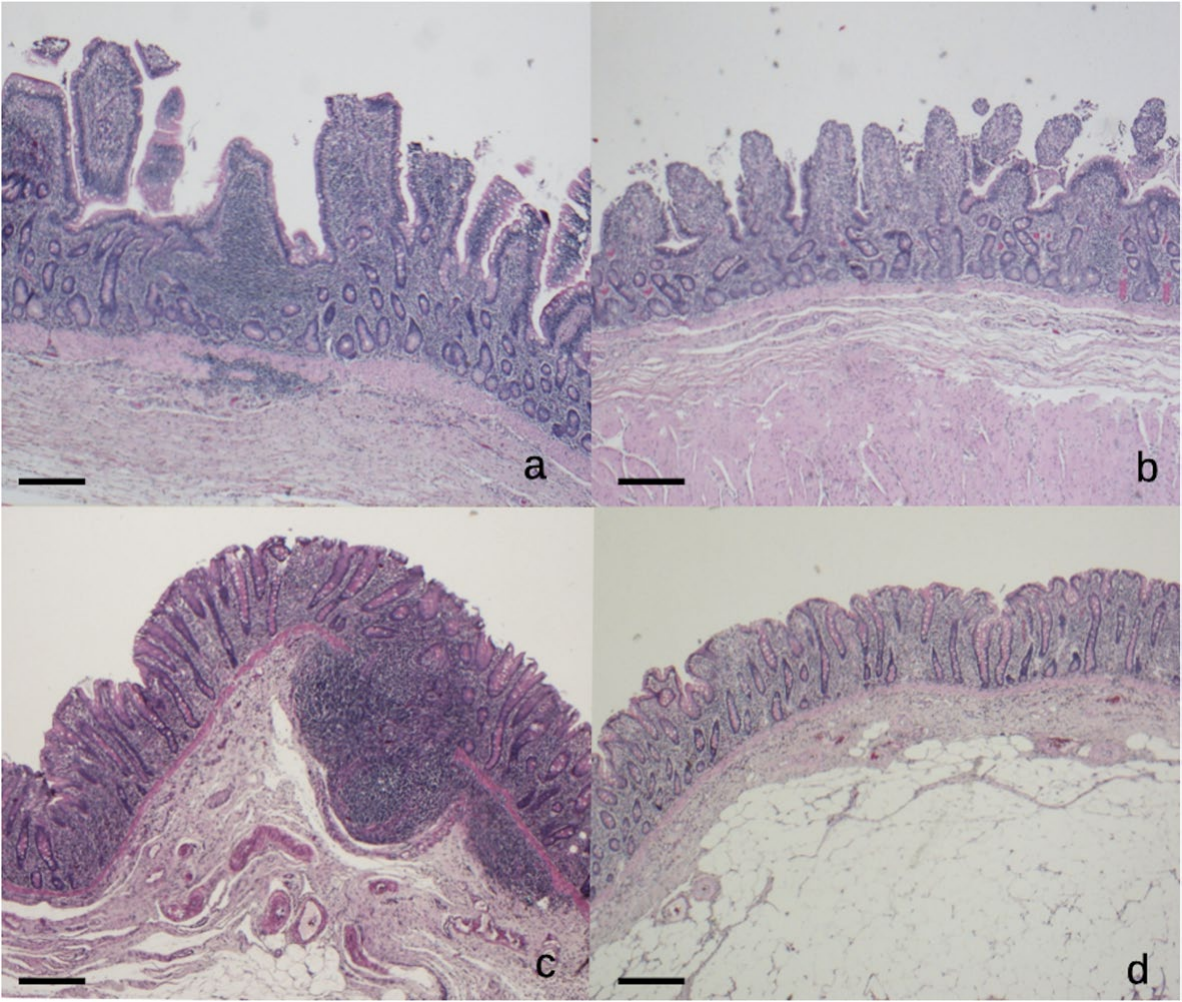
**A** Jejunum, HS group. Moderate and disseminated lymphoplasmacytic enteritis (grade 2). H-e, 2.5x. **B** Jejunum, HF group. Mild and multifocal lymphoplasmacytic enteritis (grade 1). H-e, 2.5x. **C** Pelvic flexure, HS group. Moderate and disseminated lymphoplasmacytic colitis with GALT activation (grade 2), H-e, 2.5x. **D** Pelvic flexure, HF group. Mild and multifocal lymphoplasmacytic colitis (grade 1). H-e, 2.5x

On the contrary, in the jejunum and in the pelvic flexure inflammation was influenced by diet, being more severe in HS than in HF group (*P* = 0.024 and 0.052, respectively) (Table 3) (Fig. 2A-D). In the other evaluated gut segments, non-significant differences were recorded between diet or sex (*P* > 0.05).

Regarding liver, mild and multifocal lymphoplasmacytic inflammation and multifocal to diffuse glycogenosis (mild to marked PAS staining intensity) of the hepatocytes were recorded in both groups and in both sexes. No lipid accumulation was detected (absence of Sudan Black positivity). However, diet and sex did not influence the severity of the hepatic lesions (*P* > 0.05) (Table 3).

## Discussion

To the authors’ knowledge this is the first study describing the effects of feeding management based on high amounts of starch vs. feeding management based on high amounts of fibre on several aspects of gut health in horses: severity of gastric mucosa lesions, gut histomorphometry and liver histology. Gastrointestinal health is strictly linked with the feeding management of animals and it has been proven to play a key role in guarantying animal’s health and productivity [11].

Our findings showed that the HS diet caused more severe gastric mucosa lesions in the glandular region of the stomach and more severe inflammation in the jejunum and pelvic flexure without impairing liver histology and gut morphometry.

The horses belonging to the HS group showed more severe lesions in the stomach glandular mucosa compared to HF group. Moreover, a statistical tendency was also found for the squamous region. These findings are in agreement with the current scientific literature since the higher amounts of starch supplied with the HS diet has been demonstrated to be an important risk factor for the onset of gastric ulcerations, especially in the squamous region [17]. In fact, it is reported that feeding starch-rich concentrate feedstuffs require less chewing activity and saliva production, reducing the saliva buffering of gastric acids [13, 14]. On this basis, damage to the squamous region of the stomach, known as equine squamous gastric disease (ESGD), is more likely to occur [17]. Although limiting grain intake and increasing pasture turnout seems to limit also the equine glandular gastric disease (EGGD), glandular mucosa is constantly exposed to hydrochloric acid, and unlike the squamous epithelium, has a number of protective factors to prevent mucosal damage. Therefore, it has been proposed that the breakdown of protective factors (e.g., mucus production, cytokines production) rather than exposure to hydrochloric acid alone, may be a key factor in the development of EGGD. Particularly, this can occur as a consequence of increased stress or sensitivity to stress [17]. The horses of the present study were intensively managed and this can increase the level of stress which negatively affect the horse’s physiological and behavioural needs [2]. Thus, stress due to farming conditions combined with the lack of an adequate amount of fibre could explain the more severe lesions observed in the glandular region of HS horses’ stomachs.

Considering gut morphometry, diet did not influence morphological indices of duodenum, jejunum and ileum. This is in accordance with the study of Wambacq et al. [18] that did not find any effect on small intestine morphometry after dietary supplementation of butyrate in horses. In other species, changes in the diet composition have been demonstrated to be able to modulate gut morphometry [19, 20]. This could indicate that findings in other species (e.g. poultry and piglets) may not be translated directly to the equine species, as this specific hindgut fermenter’s gastrointestinal tract shows unique characteristics [21]. Moreover, Vh/Cd was greater in the jejunum of colts (*P* = 0.037) and Cd depended on the interaction sex and diet, being greater in HS males (*P* = 0.029). Wambacq et al. [18] also observed in their study a sex-dependent effect on crypt depth in right dorsal colon. However, these results remain difficult to explain as the role of sex on gut morphometry has been scarcely studied until now, especially in horses.

As for gut histology, a lymphoplasmacytic infiltration was recorded in all the evaluated gut segments of both groups. This can be related to the stress the intensive farming system put on the horses of the present study. In fact, high stocking density and low space allowance may affect the horse’s well-being leading to stress [22]. There is increasing evidence that the gastrointestinal tract responds to stress hormones by synthesizing cytokines, hormones, and neurotransmitters which might modify microbiota diversity and increase pathogen colonization [23]. The resulted dysbiosis could explain the presence of mild to moderate lymphoplasmacytic enteritis in all the horses in the present study. However, in the jejunum and in the pelvic flexure, inflammation was higher in HS group than in HF group (*P* = 0.024 and 0.052, respectively). Thus, these findings may be due to the high amounts of starch fed to horses in the HS group [24]. In fact, when the amount of starch in the diet overcomes the small intestine physiological capacity to digest and absorb it, it will reach the large intestine where it rapidly promotes bacterial multiplication and overgrowth of acidophilic Streptococci and Lactobacilli. This can lead to lactic acidosis which provokes a decrease in luminal pH from nearly neutral to as low as 6.0, markedly impairing the survival of normal fibrolytic bacterial species (*Ruminococcaceae*) and causing dysbiosis [16, 25]. As a consequence, dysbiosis can cause inflammation associated with a higher risk of colic and diarrhoea, impairing horse’s growth performances [6]. Moreover, the proximo-distal increasing gradient recorded in the severity of gut lesions could be attributed to the fact that horses are hindgut fermenters and the majority of nutrient digestion and absorption take place in the large intestine which is more prone to develop dysbiosis and inflammation [15].

Independently from the observed stomach and intestinal lesions, no clinical signs of colic or gastric ulcerations were recorded during the trial. However, as already reported in a previous study on horses with a similar feeding management [2]– also the horses of the present study showed faeces not formed.

To prevent all of these gastrointestinal disorders, it has been suggested to not feed horses more than 2 gr/starch/kg BW/meal [6]—thus, not more than 1 kg of starch/meal for a 500 kg horse. According to Raspa et al., [4], HS group received 4 kg/animal/meal of the starch-rich complementary feed corresponding to 1.98 kg of starch/horse/meal. Therefore, the amounts of starch fed to the horses in HS group was almost two times higher than the safe level and this may be the cause of the more severe mucosa gastric lesions as well as higher inflammation observed in the jejunum and pelvic flexure of the horses belonging to the HS group. Moreover, the sex of the animals influenced the severity of inflammatory infiltrates in the duodenum and in the right dorsal colon. In particular, it was greater in females than in males (*P* = 0.024, duodenum and *P* = 0.050, right dorsal colon). Interestingly, at the end of the trial the mean ± SD slaughter bodyweight (sBW) was 347.67 ± 6.71 kg for the HS group and 344.40 ± 2.91 kg for the HF group. According to the results previously published in Raspa et al. [4], the HS diet did not result in higher BW gains or better meat quality traits than the HF diet. Also, no differences in the sBW and in the average daily bodyweight gain were found according to diet, sex or their interaction. This finding was in agreement with the study carried out on Thoroughbred (TB) yearlings by Moore_Colyer et al. [26] Indeed, they showed that TB foals fed with a fibre or a cereal-based ration achieved a similar weight gain. These results suggests that the fibre-based feeding management of horses reared for meat should be taken into account both from a welfare and an economic point of view.

The effect of sex on gut inflammation has not been investigated in horses until now. However, Kim et al. [27] suggested that sex is an important variable affecting microbiota composition both in humans and animals, probably because of the effect of sex hormones. Shift in the microbiota composition can predispose females to a greater susceptibility to suffer from dysbiosis which is capable to cause gut inflammation with the mechanisms explained above. This theory can partially explain also the differences recorded in the present study for gut morphometry between males and females as gut microbiota seems able to indirectly influence gut morphometry too [28].

Regarding liver samples, no differences between the two groups of horses were found on liver inflammation and degeneration according to the diet, sex or their interaction. Inflammation can be due to the so-called “gut-liver axis”. In fact, the liver is connected to the intestine through the portal vein, biliary tract, and numerous signalling molecules. As a result, intestinal dysbiosis can cause bacterial translocation through the portal circulation and liver sinusoids, promoting liver inflammation [25]. According to the results published in a previous study [4], it was found that the HS diet led to an increased intestinal permeability and, as a consequence, to a higher bacterial translocation. In particular, the total mesophilic aerobic bacteria counts were significantly higher in the mesenteric lymph nodes and liver samples of HS compared to HF. Although higher microbiological contamination was found in the liver of horses belonging to HS, also in the liver samples of horses belonging to HF a certain level of microbiological contamination was observed. This condition may explain the hepatic inflammation observed in all the animals. It is also important to clarify that, although diet is recognised as one of the main factors that may damage the intestinal barrier function causing leaky gut and bacterial translocation [12], other stressful conditions – e.g. intensive management – can be responsible for hepatic degeneration as a consequence of an excess of endogenous glucocorticoids, induced by the stress these horses experienced in their rearing conditions [29].

## Conclusion

This is the first study that evaluated the effect of two feeding managements, one based on a high-starch diet and the other based on a high fibre diet on several aspects of gut health in horses: severity of gastric mucosa lesions, gut histomorphometry and liver histology. The obtained results demonstrated that high amounts of starch in the horse’s diet are associated with the presence of more severe mucosa lesions in the glandular region of the stomach and a higher inflammation both in the jejunum and pelvic flexure. The present study can represent a starting point for further investigations on gut health in horses. In particular, further studies should focus on the effects of diet on microbiome composition and the role of sex in influencing horse’s gut health.

## Methods

### Animals, diets and growth performances

The experimental protocol was designed according to the guidelines of the current European Directive (2010/63/EU) on the care and protection of animals and approved by the Ethical Committee of the University of Turin (Italy) (Prot. N. 2202/2019).

The study was conducted in one of the biggest horse breeding farms for meat production in North-west Italy. The features of the farm are described in Raspa et al. [2, 30]. Briefly, a total of 19 Bardigiano horses (12 fillies and 7 colts) destined for slaughter and aged 14.3 ± 0.7 months (mean ± SD) were involved in the study. Prior to the study, all horses were dewormed using an oral gel preparation containing 200 µg/kg BW ivermectine and 1 mg/kg BW praziquantel (Equalan Duo, Merial Animal Health). After two weeks of acclimatation in an external paddock, the horses were randomly allotted to two group pens: i) a high-starch group (HS-5 fillies and 4 colts; mean ± SD initial bodyweight [iBW] 217.56 ± 9.28 kg); ii) a high-fibre group (HF-7 fillies and 3 colts; mean ± SD iBW 221.10 ± 5.00). The horses were weighted at the start and at the end of the trial and the average daily bodyweight gain was calculated according to Raspa et al. [4]. The housing and management features of the two group pens have been previously published in Raspa et al. [4]. Both groups received the same first-cut meadow hay but different complementary feeds. The complementary feeds were individually supplied twice a day (7 am and 6 pm) and gradually increased as described in Raspa et al. [4], to reach the final amount during the last 72 days of the fattening period. Horses belonging to the HS group received 8 kg/animal/day of the starch-rich complementary feed (chemical composition, as fed: crude protein 14.21%, ether extract 3.69%, crude fibre 4.44%, ash 8.30%, starch 49.50%). Horses belonging to the HF group received 3.5 kg/animal/day of the fibre-rich complementary feed (chemical composition, as fed: crude protein 19.77%, ether extract 5.06%, crude fibre 11.53%, ash 10.78%, starch 19.11%). Horses belonging to HS were fed the conventional breeding diet according to the intensive feeding management system. Instead, the HF diet was planned according to the nutritional requirements of the French “Institute National de la Recherche Agronomique” (INRA) [31]. The hay was provided at the same time (7 am and 6 pm) and estimated to be fed 6 kg/animal/day for HS and 8 kg/animal/day for HF. The overall daily nutritional composition of the diets (HS and HF) is reported in Table 4. At the end of the fattening period (129 days), horses were slaughtered according to the European Union regulations (EU Regulation 2009/853 and EU Regulation 627/2019).

**Table 4:** Nutritional composition of the total daily diets (high starch and high fibre diets): hay plus complementary feed. Adapted from Raspa et al., [4]

	**High starch diet (HS)**	**High fibre diet (HF)**
kg hay/horse/day	6.0	8.0
kg complementary feed/animal/day	8.0	3.5
Dry matter intake (kg/day)	12.6	10.3
Net Energy (MJ/day)	95.8	53.6
Crude protein (g/day)	1557.2	1159.6
Digestible Crude Protein (g MADC/day)	1177.7	723.3
Crude fat (g/day)	285.4	192.7
Starch (g/day)	3960	669
(g/kg BW/meal)	5.70	0.97
Calcium (g/day)	377.8	108.2
Phosphorus (g/day)	188.6	35.8
Lysine (g/day)	48.00	76.5
Vitamin E (mg/day)	399.7	1105.0
Selenium (mg/day)	0.48	1.72

### Stomach macroscopical evaluation

At slaughter, stomachs were isolated from the intestine, opened by cutting along the great curvature, emptied and gently washed with cold tap water in order to remove all the content. Squamous and glandular regions were evaluated separately for ESGD and EGGD, respectively. Lesions were scored from 0 to 4. In particular, the squamous mucosa was scored according to the European College of Equine Internal Medicine Consensus Statement [32]. Whereas, the glandular gastric mucosa was scored according to the scoring system proposed by Vondran et al. [33].

### Morphometrical and histopathological investigations

At slaughter, samples of duodenum, jejunum, ileum, apex of the caecum, sternal flexure, pelvic flexure, right dorsal colon and rectum were excised and flushed with 0.9% of saline solution in order to remove all the content. Samples of liver were also collected. All the samples were fixed in 10% buffered formalin and after 10 days they were routinely embedded in paraffin wax blocks, sectioned at a 5-μm thickness, mounted on glass slides and stained with Haematoxylin & Eosin (HE). Morphometric analyses were performed on HE cross-sections of duodenum, jejunum and ileum using a computerised image analysis system (Image®-Pro Plus software, 6.0 version, Media Cybernetics, Maryland, USA) coupled to a Nikon DS-Fi1 digital camera (Nikon Corporation, Minato, Tokyo, Japan) and a light microscope with a 2.5 × objective lens. The evaluated parameters were: villus height (Vh, from the tip of the villus to the crypt), villus width (Vw, across the base of the villus, but not including the brush border), crypt depth (Cd, from the base of the villus to the sub- mucosa) and mucosa thickness (form the tip of the villus to the muscularis mucosa). Villus height-to-crypt depth (Vh/Cd) ratio and the villus absorptive surface area (2π × Vh × (Vw/2) were also calculated [34]. Morphometric analyses were performed on 10 well-oriented and intact villi and 10 crypts for each intestinal segment while mucosa thickness was measured in triplicate. Additionally, all the sampled organs were submitted to histopathological evaluation using a semi-quantitative score (0: absent; 1: mild and multifocal; 2: moderate and disseminated; 3: severe and diffuse). The following histopathological alterations were investigated: inflammation and/or lymphoid tissue activation in the gut and hepatocyte degeneration and lymphoid tissue activation/inflammation in the liver. In order to investigate the accumulation of lipids and glycogen in the liver, frozen and paraffin embedded tissue samples of this organ were also stained with Sudan Black and Periodic acid-Schiff (PAS), respectively. All the slides were blindly evaluated by three different observers and the discordant cases were reviewed using a multi-head microscope until a unanimous consensus had been reached.

### Statistical analysis

Statistical analysis was conducted using R software version 4.0.4 (R Foundation for Statistical Computing, Vienna, Austria; http://www.r-project.org). The Shapiro–Wilk test was used to test the normality of the data distribution and a robust Anova test was performed by the trimmed means method. The ANOVA test allowed the evaluated variables to depend on three fixed factors (diet, sex, and the interaction between diet and sex). The interactions were evaluated by pairwise comparisons. Data were described as mean and standard deviation (SD). *P* values ≤ 0.05 were considered statistically significant.

## Data Availability

The datasets analyzed in the present study are available from the corresponding author on reasonable request.
